# Tunable Ultra-high Aspect Ratio Nanorod Architectures grown on Porous Substrate via Electromigration

**DOI:** 10.1038/srep22272

**Published:** 2016-02-29

**Authors:** Ali Mansourian, Seyed Amir Paknejad, Qiannan Wen, Gema Vizcay-Barrena, Roland A. Fleck, Anatoly V. Zayats, Samjid H. Mannan

**Affiliations:** 1 Department of Physics, King’s College London, Strand, London WC2R 2LS, UK; 2 Department of Physics and Materials Science and Centre for Functional Photonics (CFP), City University of Hong Kong, Tat Chee Avenue, Kowloon, Hong Kong; 3 Centre for Ultrastructural Imaging (CUI), King’s College London, New Hunt’s House, Guy’s Campus, London, SE1 1UL, UK

## Abstract

The interplay between porosity and electromigration can be used to manipulate atoms resulting in mass fabrication of nanoscale structures. Electromigration usually results in the accumulation of atoms accompanied by protrusions at the anode and atomic depletion causing voids at the cathode. Here we show that in porous media the pattern of atomic deposition and depletion is altered such that atomic accumulation occurs over the whole surface and not just at the anode. The effect is explained by the interaction between atomic drift due to electric current and local temperature gradients resulting from intense Joule heating at constrictions between grains. Utilizing this effect, a porous silver substrate is used to mass produce free-standing silver nanorods with very high aspect ratios of more than 200 using current densities of the order of 10^8^ A/m^2^. This simple method results in reproducible formation of shaped nanorods, with independent control over their density and length. Consequently, complex patterns of high quality single crystal nanorods can be formed *in-situ* with significant advantages over competing methods of nanorod formation for plasmonics, energy storage and sensing applications.

Electromigration (EM) is defined as the transport of atoms driven by momentum transfer from electron flow inside a current carrying material. This can lead to structural changes such as whisker growth and stress induced voids^1^,^2^. Electromigration is normally depicted in a negative light, as a serious problem for Very-Large-Scale Integration (VLSI) and Ultra-Large-Scale Integration (ULSI) electronic circuits due to the increasing current densities that accompany miniaturization^3^. Therefore much effort has been directed at developing new electronic materials, wiring designs and fabrication methods so as to minimize the effects of electromigration^4^. However, electromigration has recently been used constructively as a tool for fabrication of zero and one dimensional nanocrystals^5^ nanostructures for local electric field enhancement in plasmonics^6^,^7^, molecular-scale biochemistry measurements^8^,^9^,^10^,^11^ and to control the kinetic faceting of surface orientations that belong to the equilibrium shape of the crystals^12^. In general electromigration is affected by a large number of parameters such as current density, temperature, film thickness, grain size and timescale^1^,^2^,^3^,^4^,^13^. Previous attempts to create whiskers using electromigration either resulted in whiskers growing only at the anode^14^,^15^ or required precise local conditioning of the substrate to generate localised whiskers^15^,^16^. An industrial method with control over mass production of whiskers over an entire substrate would have applications in plasmonics^17^, energy storage^18^ and sensing applications^3^,^4^,^6^,^7^ due to the high aspect ratio and large surface area structures that could be produced.

In this work, we demonstrate that electromigration can be applied to grow a dense structure of nanorods on a porous Ag substrate rather than the sparse nanorod formation previously observed. Electromigration in a porous medium is also shown to result in nanorods being formed along the length of the conductor rather than being confined to the anode as in a typical non-porous media. In addition to the high density of nanorod formation, it is found that the density and nanorod length can be independently controlled. The ability of the process to produce single crystal nanorods with aspect ratios exceeding 200 is also highly noteworthy, as is the production of high aspect ratio platelets in addition to nanorods. Furthermore, electromigration in a porous medium results in transformation of the internal pore and grain structure, an effect which has not previously been reported but which may have interesting technological applications in its own right. The growth mechanism of nanorods along the length of the conductor can be explained by the interaction of the normal electron wind force^19^ driving atoms from cathode to anode with thermal gradients generated by the presence of the pores causing current constrictions. The simplicity of nanorod formation by electromigration, utilizing voltages ~7 mV across the ~500 μm length of the conducting stripe to generate high current density, may have significant advantages over other methods of nanorod growth^20^ and in particular may allow complex patterns of nanorods being grown simply by controlling the local current densities.

## Results

**Figure 1:**
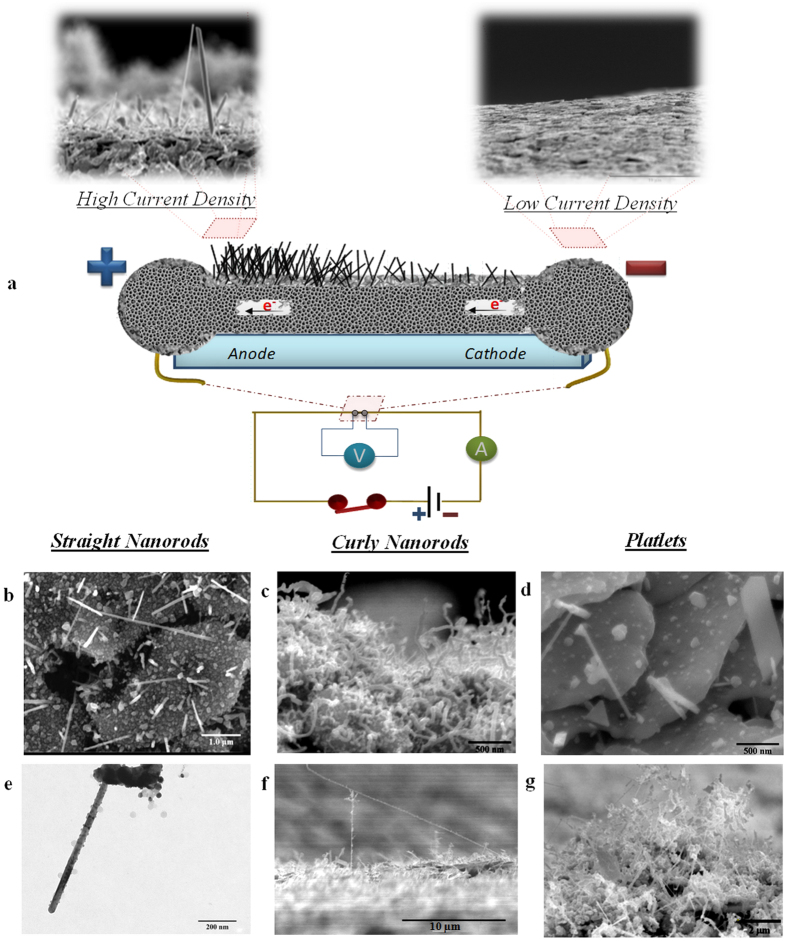
Schematic of experimental setup and different types of nanorod fabricated via the EM process. (**a**) Schematic diagram of porous sintered silver stripe and the EM experimental setup. SEM image top left shows side view of nanorods at high current density regions (2.34 × 10^8^ A/m^2^), and SEM image top right shows a low current density region after 240 hours. (**b**) SEM micrograph of straight silver nanorods under current density of 2.45 × 10^8^ A/m^2^ after 240 hours (uninterrupted). (**c**) SEM micrograph of curly nanorods under current density of 2.24 × 10^8^ A/m^2^ (uninterrupted) for 480 hours. (**d**) SEM image showing mix of nodules, nanorods and platelets at current density of 2.34 × 10^8^ A/m^2^ after 240 hours (interrupted). (**e**) TEM image of a typical 20 nm diameter nanorod. (**f**) SEM image of nanorod with high aspect ratio ~200 generated on the substrate after 240 hours continuous EM. (**g**) SEM image of the nanorods growing until their density is high enough to meet neighbouring nanorods.

A schematic of the EM experimental setup is shown in Fig. 1a. The nanorod density, location, size and diameter are controlled by EM duration, current density and interruptions. EM was carried out on five samples (see Methods and Supplementary information) in air, at two temperatures (ambient and 200 °C) with current densities ranging from 2.2 × 10^8^ to 2.45 × 10^8^ A/m^2^ (Table S1). The duration of the electromigration processes was from 6–480 h. Electron microscopy (SEM and TEM) analysis reveals high quality single crystal nanorods with diameter down to 20 nm (Fig. 1b,e). Straight nanorods occur in short duration EM experiments of up to 240 h (Fig. 1b). Figure 1c shows constant diameter curly nanorods formed after long duration uninterrupted EM. Platelets with hexagonal tips (Fig. 1d) can also be formed as a result of the initial protrusions expanding followed by growth. The hexagonal shape indicates that the rod emerges from a [1, 1, 1] plane of the fcc Ag lattice^21^,^22^. The EM experiments running for 480 h (Fig. 1c,g) show that the nanorods continue to grow until the density of the generated nanorods is high enough for them to meet neighbouring nanorods which may be responsible for limiting nanorod growth to a maximum length of ~20 μm (Fig. S1b–d). Figure 1g shows a high density of nanorods with possible instances of welding between them. Previous studies have reported welding of individual silver nanorods under high current density at the point of contact^23^. Increasing current density to 1.70 × 10^9^ A m^−2^ results in amalgamation of grains and pores at the cathode where reduction in the number of conduction paths leads to high localised heating and circuit failure after a short time of 18 h (Fig. S2). Increasing temperature up to 200 °C in samples (for 120 and 240 h) does not result in nanorod formation after EM.

**Figure 2:**
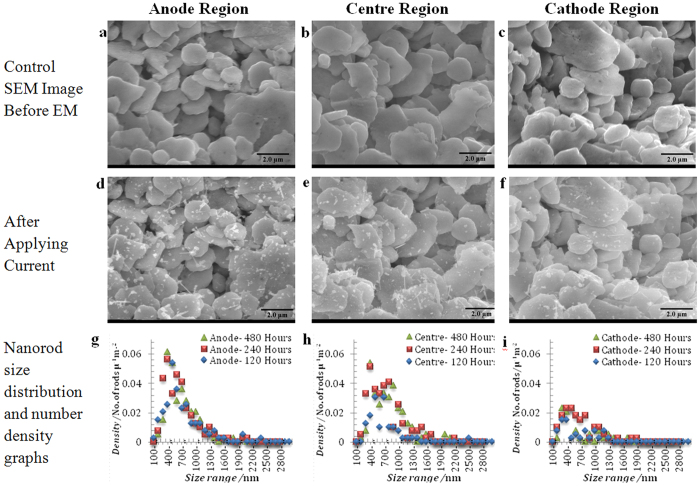
Comparison of nanorod formation in anode, centre and cathode regions. Comparison of size distribution and number density of nanorods on the sintered silver stripe taken (**a**–**c**) before and (**d**–**f**) after applying current at (**a**,**d**) anode, (**b**,**e**) centre of stripe and (**c**,**f**) cathode under current density of 2.45 × 10^+8^ A/m^2^ after 240 hours. The graphs (**g**–**i**) represent the number density of nanorods versus their length at the anode (**g**), centre (**h**), and cathode (**i**) regions of the corresponding images after 120, 240, and 480 hours, respectively.

Figure 2 shows a comparison of the top surface of the Ag stripe S3 before and after EM for 240 h, at the anode, centre and cathode. The number density of nanorods decreases steadily between anode and cathode, with the density of nanorods at the cathode approximately one third that of the anode. This suggests that a uniform coverage of nanorods might be achieved by simply reversing the flow of current so that both ends of the stripe spend equal times as anode and cathode. Figure 2a–f shows the change in nanorod size distribution and number density across the stripe. Figure 2g–i summarizes the changes in number density and average nanorod length (not adjusted for orientation) across sample S3 for time periods of 120, 240 and 480 h. The average length of the nanorods ranges from 400 to 550 nm and longer nanorods do still appear across the sample, with the largest rods reaching an apparent length of 20 μm (Fig. 1f). Nanorod diameter is relatively constant at 25–40 nm, with some larger diameter rods (up to 100 nm) produced in one sample S2 (Table S1) which experienced multiple connection and disconnection events.

**Figure 3:**
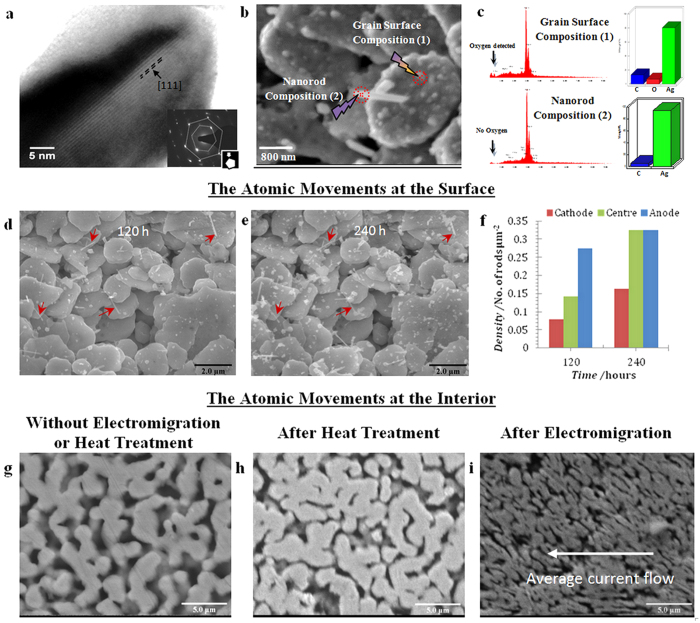
Characterisation of generated nanorods and the effect of the oxide layer. (**a**) TEM image of a single 40 nm diameter nanorod. The selected area electron diffraction (SAED) pattern indicates a standard FCC structure interpreted as the overlapping of the [111] and [110] zone axes^21^. The orientation of nanorods with respect to the electron beam is shown in the inset of image. (**b**,**c**) EDX spectral analysis at the positions marked with crosses on the SEM micrograph (**b**) from sample with current density of 2.45 × 10^8^ A/m^2^. Spectrum (1) shows oxygen on the substrate indicating an oxide layer whereas the nanorod spectrum (2) shows no oxygen. (**d**) Formation of nanorods after EM for 120 hours. (**e**) Disconnection and reconnection followed by another 120 hours EM; red arrows indicate the nanorods formed after the first 120 hours which all showed zero growth after reconnection. (**f**) Total nanorod number density before and after disconnection/reconnection event. (**g**–**i**) SEM micrographs of the internal structure of sintered silver (**g**) before EM, (**h**) under 200 °C heat treatment for 480 hours showing no structural change and (**i**) after EM for 480 hours with a current density of 2.34 × 10^8^ A/m^2^ showing massive EM driven grain restructuring.

Characterisation of the fabricated nanorods shows the formation of good quality single crystal nanorods at the top surface of the samples. The TEM image of the 20 nm diameter nanorod is shown in Fig. 3a,b and the Selected Area Electron Diffraction (SAED) patterns^22^,^24^ are interpreted as the overlapping of [111] and [110] zone axes which indicate a six fold symmetry previously associated with silver nanorod growth. The orientation of nanorods with respect to the electron beam is shown in the inset of (Fig. 3b). Previous studies have suggested that weak points in a metal oxide layer act as nucleation sites for nanorod growth^25^. Figure 3c shows Energy Dispersive X-Ray (EDX) analysis on a nanorod and on a grain at the surface of the stripe. The bar chart shows that at the surface of the sintered silver grains after electromigration the composition contains 8% oxygen whereas the composition is 0% oxygen in the spectrum taken from the nanorod. These results suggest the existence of a thin oxide layer on the surface of the silver stripe during the EM process. In order to test this hypothesis further, EM on sample S3 (Fig. 3d,e) was interrupted after 120 h for a period of 240 h and then restarted with the same current density as before for an additional 120 h. The original rods (indicated by the red arrows) did not continue growing after the interruption, but new rods emerged, as shown by Fig. 3f. This behaviour was repeated at other locations and in other samples, suggesting that new weak spots in the thin oxide layer form when the samples cool down and contract during the interruption. The spectrum of nanorods after multiple interruption (Table S1) shows evidence of an oxide layer forming on the original nanorods which could account for the lack of growth in these nanorods (Fig. S4).

While there is no change in surface grain morphology during EM evident from either Fig. 2 or Fig. 3 apart from nanorod growth, massive change in internal grain structure is seen in Fig. 3g,i. This is not purely a result of thermal effects as shown by the control (Fig. 3h) which was stored at 200 °C for 480 h while the sample of Fig. 3i was subjected to EM. It should be noted that the structure seen in Fig. 3i after EM was found throughout the stripe at anode, centre and cathode and that the orientation of the elliptical grains changes throughout the stripe and does not appear to be simply correlated with the cathode-anode axis (arrow, Fig. 3i). The contrasting surface and interior behaviour indicates that while the oxide layer at the stripe surface prevents atomic migration along grain surfaces, in the stripe interior, grain surfaces do not support an oxide layer that prevents surface diffusion. Voids have not been directly detected at the surface of the porous sample, but formation of nanorods is accompanied by an increase in resistance (1–5%), followed by rapid resistance fluctuations and finally open circuit, accompanied by a crack. This can be explained with reference to Fig. 3g,i where it is seen that significant internal transformation of structure is occurring. Calata *et al.*^26^ also observed formation of cracks at locations where the current density increases abruptly and attributed this to high atomic flux density in those regions.

Thermal gradients are known to affect the divergence of the atomic flux and hence void and hillock (or nanowire) formation in EM experiments due to the dependence of electrical resistivity with temperature^13^,^27^. In order to calculate the magnitude of thermal gradients, electrical resistivity measurements of the stripe were performed using the four point probe method as the current was varied and the temperature in the stripe calculated using the coefficient of electrical resistivity variation with temperature. As an example, it was found that a current density of 2.4 × 10^8^ A/m^2^ resulted in a temperature rise of 83 °C.

**Figure 4:**
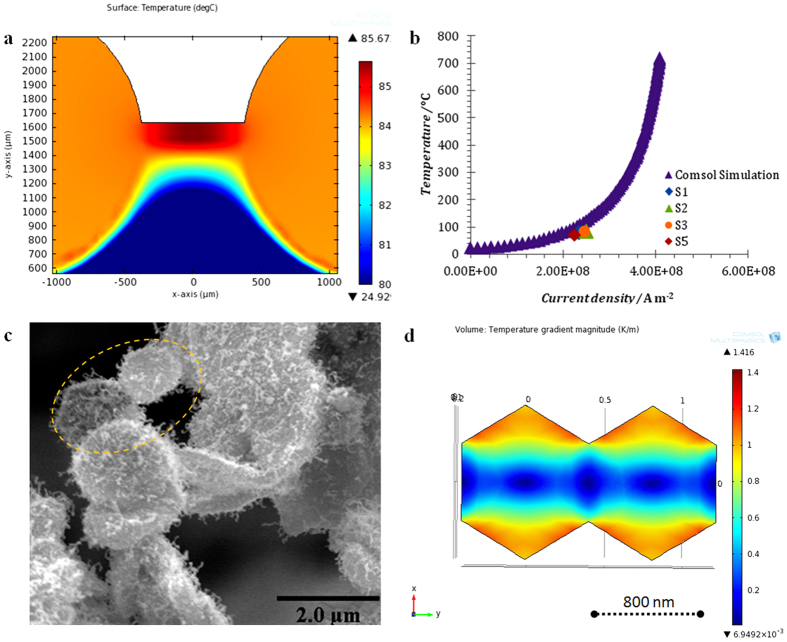
Temperature distribution at stripe and grains. (**a**) Temperature distribution within the stripe found using FEM simulations, (**b**) Experimental and simulated temperature at the centre of the stripe. (**c**) SEM image of silver grains for sample S3. (**d**) FEM simulations of the temperature gradient magnitude in two neighbouring grains similar to those highlighted by the dotted line in (**c**).

The experimental determination and modeling of temperature distributions at the scale of the stripe and the scale of individual grains are shown in Fig. 4a,b. The temperature calculations for all samples from experimental results are in good agreement with temperature calculations using the Finite Element (FEM) model (details in Methods). SEM images of porous silver (Fig. 4c) have been used to construct a simple numerical model of current flowing between grains via a constriction and the resulting temperature gradients are shown in Fig. 4d. The results indicate high values of temperature gradients at the outer edges of the grains and especially near the constrictions. The atomic flux divergence (accumulation rate of atoms) is proportional to the dot product of atomic flux density and the temperature gradient^13^,^27^,^28^, 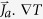
and hence the numerical simulations (Table S2) can be used to estimate the rate of atomic accumulation due to the local temperature variations at grain level in order to test the hypothesis that these variations, together with the stripe level variations (Fig. 4a) are responsible for the observed pattern of nanorod growth across the stripe.

The number of atoms deposited on a grain surface is estimated from the volume of nanorods in SEM images taken from two similar sized grains after two consecutive EM time periods of 120 h. The experimental results are then compared with the atomic flux divergence model. We have taken the temperature gradient value from FEM simulations to calculate the Atomic Flux Divergence (AFD) using the portion of atomic flux from Eq. 3 due to electromigration, 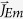
1,25,28:


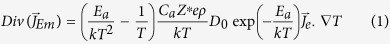


**Figure 5:**
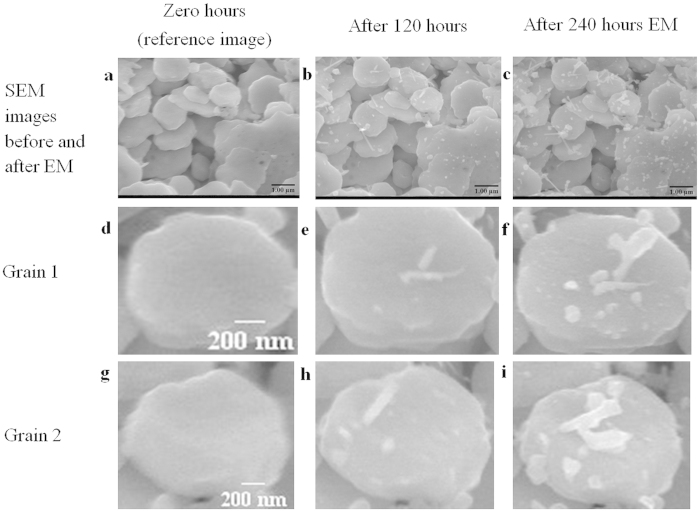
Source images for Atomic Flux Divergence (AFD) calculation in Table 1 of number of atoms deposited in two similar sized grains. SEM images of the anode region of sample S3 (**a**) before EM, (**b**) after 120 hours and (**c**) after 240 hours at the same location. Two similar sized grains (Grain 1 (**d**) and 2 (**g**)) were selected to compare the calculation of atomic deposition numbers after 120 hours (**e**,**h**) and after 240 hours (**f**,**i**).

The value of the AFD (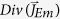), representing the number of atoms deposited per unit volume and in unit time, has been calculated as 3.46 × 10^+19^ atom/m^3^/s using the values in Table S2 as an input. Multiplying the AFD by the volume of the grain and timescale of EM (120 h and 240 h) results in an estimate of the number of atoms deposited in a grain and is compared to values measured from SEM images in Fig. 5 experimentally in Table 1.

## Discussion

The anomalous Ag nanorod formation at anode and central locations can be explained by the presence of complex thermal gradients generated by Joule heating in the constrictions between grains. An analysis based on Fick’s laws of diffusion shows that atoms should accumulate in high current density regions where thermal gradients exist (See Methods). This calculation has considered only lattice diffusion in order to give a lower bound, explaining the discrepancy in Table 1. A detailed calculation including grain boundary diffusion should result in closer agreement between the measured and calculated values and will be the subject of further work. However, the present work shows that the proposed mechanism of thermal gradients operating over the length scale of individual grains in combination with electromigration being responsible for nanorod growth is reasonable.

Within the interior of the sample, fast diffusion along the grain surfaces facilitates the internal grain refinement observed in (Fig. 3i) while at the surface the oxide layer prevents rapid surface diffusion and allows compressive stresses to build up locally, eventually leading to the observed nodule (nanodot) and nanorod formation at weak spots in the oxide layer. We note that in a porous material therefore, electromigration can be used to probe the chemical composition at the surface of the internal pores and in particular the absence or presence of an oxide layer inhibiting diffusion. The Ag oxide layer is key to formation of the nanorods and explains why the elevated temperature experiments failed to produce nanorods. Previously reported experimental data^29^ shows there are three phases of AgO_x_ film commonly found on a Ag surface. These are a silver rich phase (phase I), mostly Ag_2_O (phase II) and finally a mixed phase of Ag_2_O and AgO (phase III). The Phase III is the least stable and can easily decompose to Ag_2_O and O_2_ even at temperatures below 160 °C. The elevated temperature tests and the high current density experiment presumably lead to decomposition of the Phase III layer and prevention of the compressive stress build up that is a prerequisite for nanorod formation. Similarly, the interrupted EM experiments which lead to growth of new nanorods can be explained by the interruption allowing oxide to form on fresh nanorod surfaces and the thermo-mechanical stresses as a result of temperature changes leading to formation of new weak spots on the grain surfaces. The ability to modify nanorod characteristics after initial fabrication can be used to construct sensors with nanorods grown *in situ* or modified *in situ* to optimize their sensitivity.

## Conclusion

EM has been used as a constructive process leading to mass fabrication of nanorods simply by passing current through a stripe of porous material under controlled current density. Additionally, the internal grain refinement observed in the porous structure has no analogy in non-porous materials, and is facilitated by the large oxide free surface area present in these porous materials. Absence of grain refinement at the surface of the substrate, EDX measurements on the nanorods, heated samples and interrupted EM experiments all indicate that an oxide layer on the exterior Ag surface restricts atomic diffusion here and hence allows compressive stress build up leading to nanodot and then nanorod formation at weak points of the oxide layer. The mechanism of nanorod growth away from the anode in a porous substrate was investigated by atomic flux divergence calculations taking into account thermal gradients on the scale of a single grain and assuming only lattice diffusion. The calculations show that the number of deposited atoms in a grain due to thermal gradient fluctuations is an order of magnitude lower than observed experimentally but supports the hypothesis that grain-scale thermal gradients are responsible for the observed nanorods once the higher atomic flux from grain boundary diffusion is taken into account. In contrast to the exterior surface with its oxide layer constraining surface diffusion, the internal pore surfaces in sintered silver offer fast surface diffusion pathways. This results in refining of the pore structure during EM. Electromigration can hence be used as an *in-situ* probe of the surface condition of the interior pores in conducting porous media as well as a mechanism by which these pores can be transformed. Finally the experimental approach reported here suggests that high density, high aspect ratio nanorods can be fabricated using EM with precise control of nucleation density and size achievable by modulating the current density.

## Methods

### Experimental

The porous Ag samples were fabricated using NanoTach^®^ X silver paste produced by NBE Tech, a paste used for attaching semiconductor die to ceramic substrates typically for power electronics applications. The paste consists of ~30 nm diameter Ag particles together with ligands to prevent agglomeration and organic components to improve paste rheology. The paste is printed onto the edge of a glass cover slip to create a simple sample geometry with a high degree of control over parameters such as length, width and thickness. The same paste is then applied at both ends to connect gold wires at anode and cathode ends after a further sintering step. A feature of this EM setup is that the anode and cathode is not defined by junctions with a refractory metal as in a typical Blech setup^30^, but by the existence of sharp variations of cross sectional area resulting in high current density inside the stripe and low current densities beyond the anode and cathode to form a 3D analogue of the bow-tie structure^31^. Figure 1a shows a schematic of the experimental setup. Figure S1a shows a TEM image of a cross section through the sintered material revealing that the original nanoparticles have merged to form grains ~1 μm diameter with a high density of twin boundaries and a porosity of ~25% (see^32^ for further details of the structure).

### Diffusion model of atomic accumulation and Finite Element Modeling (FEM) simulations

Voids form during electromigration by depletion of atoms while nanorods grow in regions where atoms accumulate. Based on Fick’s laws of diffusion, the governing diffusion-convection equation for atomic evolution can be written as^13^,^27^,^28^


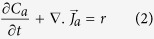


where *C*_*a*_ is the atomic concentration, 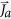 is the total atomic flux, *r* is the source/sink term which in the simplest models is given by −Δ*C*_*a*_ /*τ* where Δ*C*_*a*_ represents the excess of atoms from its equilibrium value and 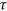 represents a relaxation time. The atomic flux vector contains contributions from self-diffusion, electric current (‘wind force’), temperature gradient and hydrostatic stress gradient shown respectively as the terms in Eq. (2) 1,25,28


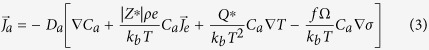


where *D*_*a*_ is the diffusivity of atoms, Z* is the effective charge, *e* is the elementary charge, *k*_*b*_ is Boltzmann’s constant, *T* is the absolute temperature, 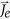 is the current density, Q* is the heat of transport, *f* is the atomic relaxation factor, Ω is the atomic volume, and 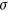 is the hydrostatic stress. *D*_*a*_ can further be expressed by Arrhenius law as


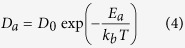


where *D*_0_ is the pre-exponential factor and *E*_*a*_ is the activation energy. Equations (1, 2, 3) form the standard base for discussion of electromigration, and have been investigated extensively for Cu and Al on-chip interconnects^13^,^28^. Numerical estimates of the temperature gradient term in Eq. (2) show that the direct effect of the temperature gradient on 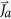 is negligible. However, the 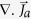 term in Eq. (1) leads to significant atomic accumulation or depletion arising from temperature gradients via the spatial variation in *D*_*a*_ given by Eq. (3) in the presence of a thermal gradient. Hence knowledge of the local current density and temperature distribution is required to calculate 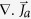 and nanorod growth.

To calculate the current and temperature distribution within the stripe we have used commercial FEM software COMSOL (Joule heating module). The electrical current has been simulated within the silver stripe from the source electrode to the drain electrode taking into account Joule heating, conduction through the glass at a current density of 2.4 × 10^8^ A/m^2^. The temperature of the bottom surface of 3000 μm thick SiO_2_ substrate on which the stripe is located was set to the room temperature of 298.15 K. The thermal Conductivity of SiO_2_ was given the textbook value of 0.8 W/mK^33^ and the thermal conductivity and electrical resistivity of material were similarly set at 250 W/mK and 7.33 × 10^−8^ Ω m respectively^34^.

## Additional Information

**How to cite this article**: Mansourian, A. *et al.* Tunable Ultra-high Aspect Ratio Nanorod Architectures grown on Porous Substrate via Electromigration. *Sci. Rep.* **6**, 22272; doi: 10.1038/srep22272 (2016).

## Supplementary Material

Supplementary Information

## Figures and Tables

**Table 1:** Comparison of the deposited number of atoms calculated both from the Atomic Flux Divergence model and experimental measurements on two similar size grains on sample S3 for two different time periods of 120 and 240 h.

Time (h)	Number of atoms (Grain 1)		Number of atoms (Grain 2)	
	Atomic Flux DivergenceModel (AFD)	ExperimentalMeasurement	Atomic Flux DivergenceModel (AFD)	ExperimentalMeasurement
120	2.37 × 10^6^	5.92 × 10^7^	2.53 × 10^6^	5.85 × 10^7^
240	4.74 × 10^6^	1.32 × 10^8^	5.06 × 10^6^	1.49 × 10^8^
