# Supplementary material for: Tunable Ultra-high Aspect Ratio Nanorod Architectures grown on Porous Substrate via Electromigration
Source: Sci Rep. 2016 Feb 29;6:22272. doi: 10.1038/srep22272 (PMC4770296; doi:10.1038/srep22272)
Supplement: Supplementary Information [file srep22272-s1.pdf]

Supplementary Information

Tunable Ultra-high Aspect Ratio Nanorod Architectures grown on Porous Substrate via Electromigration

Ali Mansourian[1], Seyed Amir Paknejad[1], Qiannan Wen[2], Gema Vizcay-Barrena[3], Roland A. Fleck[3], Anatoly V. Zayats and Samjid H. Mannan[1*]

[1]Department of Physics, King's College London, Strand, London WC2R 2LS, United Kingdom

[2]Department of Physics and Materials Science and Centre for Functional Photonics (CFP), City University of Hong Kong, Tat Chee Avenue, Kowloon, Hong Kong

[3]Centre for Ultrastructural Imaging (CUI), King's College London, New Hunt's House, Guy's Campus, London, SE1 1UL, United Kingdom

Samjid.mannan@kcl.ac.uk

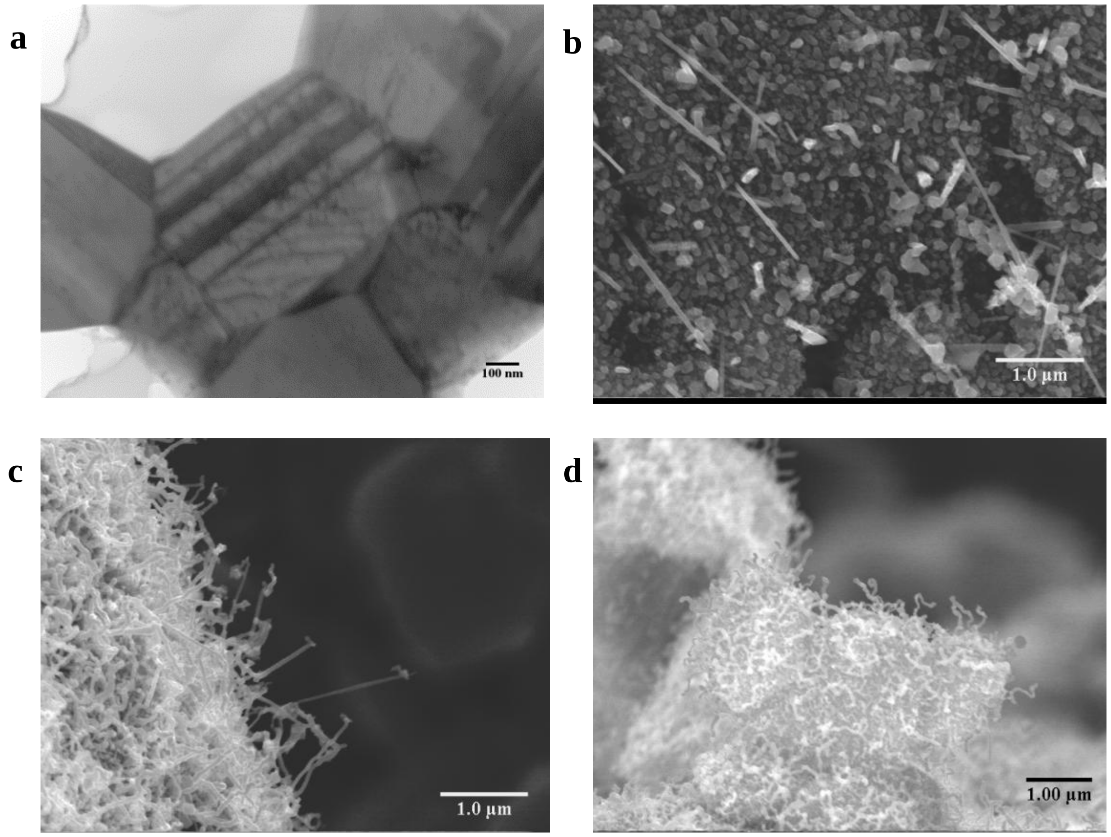

Figure S1. (**a**) TEM image of internal sintered silver microstructure before EM. (**b**) SEM image taken from the sample S3 anode region after 240 hours EM, showing linear nanorods and nodules, (**c**) the side view SEM images taken from the anode (sample S2) after 240 (with an interruption after 120 hours) showing very high density of nanorods formed long enough to meet each other, (**d**) SEM images taken from the anode side of sample S1 after 480 without any interruption of experiments showing the high density formation of curly shaped nanorods at different regimes of current density according to the local cross-section.

Details of samples

Table S1. Details of samples show the different current densities, EM duration and number of Interruptions of the samples used for experiments and the stripe temperature. Typical stripe dimensions were 500 µm length, 160 µm thick, 165 µm wide. An Iso-Tech IPS2303D power supply was used to generate ~1V across the circuit which resulted in ~ 7 mV across the length of the stripe. Rapid void formation and current constriction lead to melting of Ag in S5.

Samples	S1	S2	S3	S4	S5
Current Density (A/m^2)	$2.24 \times 10^{+8}$	$2.34 \times 10^{+8}$	$2.45 \times 10^{+8}$	$2.35 \times 10^{+8}$	$1.17 \times 10^{+9}$
EM Duration	480 hours	480 hours	480 hours	120 hours	18 hours
Number of Interruptions	None	3	2	None	None
Temperature (°C)	70.94	79.28	83.19	79.64	Melting temperature (961.8 °C)

High localised heating in S5 showing evidence of melting after 18 h in centre.

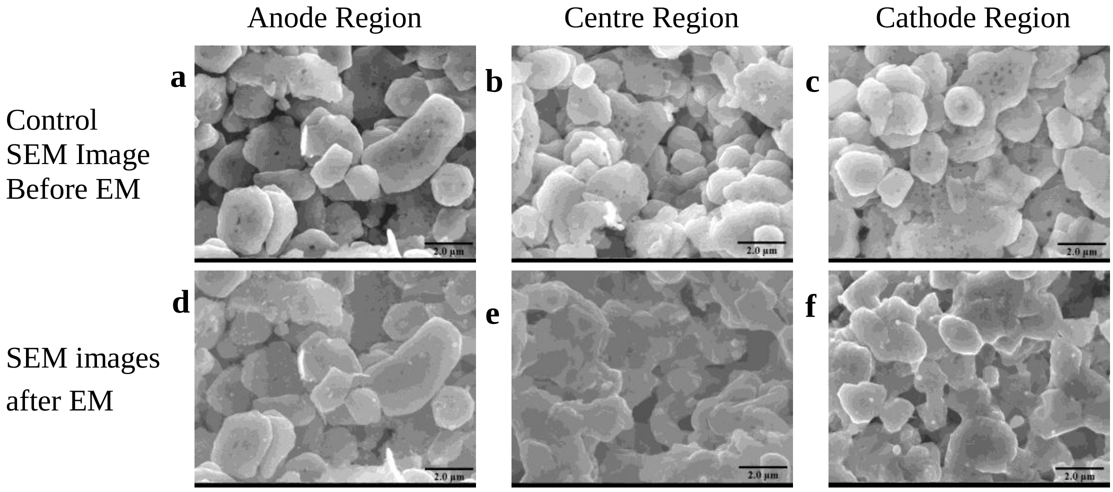

Figure S2. High localised heating in S5 showing evidence of melting after 18 hours. SEM images taken from anode, centre and cathode region on sample S5 before EM in (**a-c**) and after EM in (**d-f**) under current density of $1.17 \times 10^{+9}$ A/m^2 showing evidence of partial melting probably caused by progressive restriction of conducting pathways due to increased voiding at cathode and centre leading to intense Joule heating in these regions.

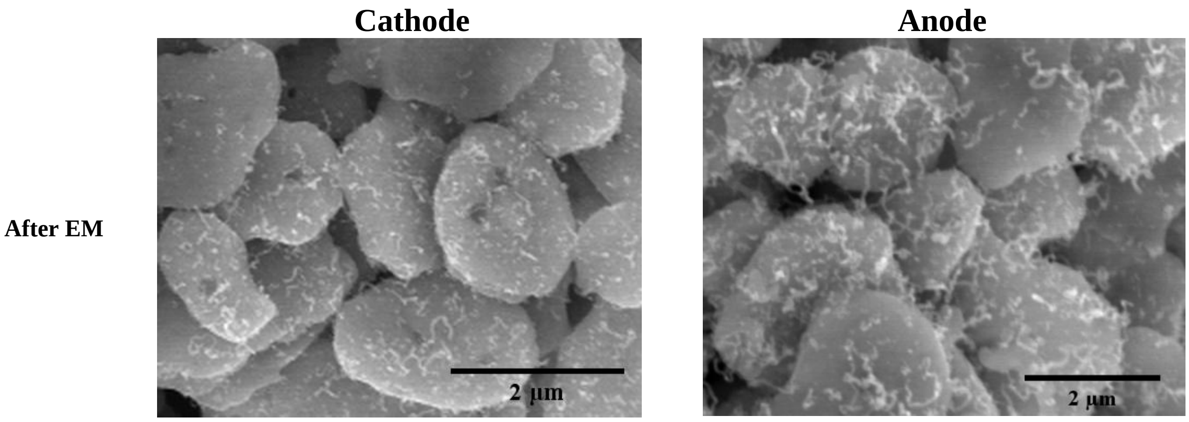

Figure S3. Showing that after a long EM period of 480 hrs the differences between (a) cathode and (b) Anode have lessened compared to smaller EM timescales.

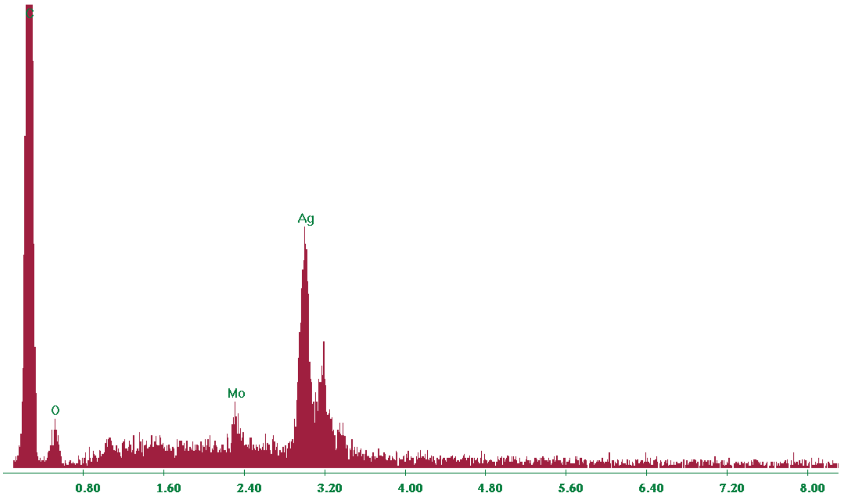

Figure S4. Shows the presence of oxygen on EDX spectra from a nanorod that experienced multiple EM interruptions.

Table S2. Parameters for diffusion–convection model

Parameters	Value	Dimension	References
∇T (Temperature difference)	1.41	K/m	COMSOL
D_0 (Diffussion coefficient)	3.95×10^{-5}	m^2/s	(*9*)
E_a (Activation energy)	7.32×10^{-20}	Joul	(*9*)
Z* (Effective charge)	-6.5	----	(*8*) and (*10*)
ρ (Resistivity)	7.33×10^{-08}	Ω m	From experiments at 80 °C
J_e (Current density)	$2.40\times10^{+08}$	A/m^2	From experiments
C_a (Atomic concentration)	$4.38\times10^{+28}$	$(atom/m^3)$	(*11*)
T (Temperature of EM)	$3.53\times10^{+02}$	K	Temperature of EM Experiments (Cal)
